# Dose-finding designs for cell therapy cancer clinical trials evaluating drug-combinations

**DOI:** 10.1016/j.cct.2025.107894

**Published:** 2025-03-30

**Authors:** Evan M. Bagley, Nolan A. Wages

**Affiliations:** a Department of Public Health Sciences, Medical University of South Carolina, Charleston, SC, USA; b Department of Biostatistics, Virginia Commonwealth University, Richmond, VA, USA

**Keywords:** Drug combinations, Dose-finding, Cancer clinical trials, Cell therapy, Late-onset toxicity, Dose-feasibility

## Abstract

Cell therapies have taken hold as a promising modality of treatment for multiple types of cancer. Despite this, their efficacy as a monotherapy has been limited, driving interest in their possible role as part of a drug combination. This paper introduces a novel dose-finding design for phase I cancer trials assessing drug combinations where one of the drugs is a cell therapy. Our design adapts the partial order continual reassessment method (POCRM) to account for late-onset dose-limiting toxicities (DLT) and feasibility concerns due to complexities related to manufacturing the cell therapy product. We illustrate our design on a proposed trial to assess the combination of a Rituximab-based bispecific antibody activated T cell product and Nivolumab for the treatment of high-grade B-cell lymphoma. We also provide simulation results that demonstrate our design’s ability to accurately identify the feasible maximum tolerated dose combination (FMTDC) while managing late-onset DLTs and feasibility concerns. Our methodology aims to improve the phase I drug combination landscape for cell therapy cancer clinical trials.

## 1. Introduction

This paper addresses the challenges encountered in phase I cancer trials that evaluate drug-combination therapies when one of the treatments includes a cell therapy product and there is a possibility of late-onset toxicity. Cell therapies represent a highly promising avenue in immuno-oncology, with a noticeable increase in active trials since at least 2018 [1]. However, when used as a standalone treatment, cell therapy products face significant limitations and uncertainties regarding their efficacy and safety [2]. Consequently, there is a growing interest in drug combination therapies, which could offer enhanced efficacy through the synergistic effects of combined treatments. Numerous studies have demonstrated that combining cell therapy with other cancer treatments, such as radiotherapy, chemotherapy, and checkpoint inhibitors, can lead to improved outcomes [3]. Despite this increased interest in cell therapy combination trials, there are issues that arise from their implementation and execution in the dose-finding setting. The three primary contemporary dose finding problems we will address are (1) some patients might not be feasible to receive their assigned dose, (2) the toxicity order of the dose levels may be only partially known, and (3) some patients may have dose limiting toxicities (DLTs) long after accrual to the study.

The first challenge, termed dose-feasibility, emerges when the cell therapy product fails to reach the desired dose level during the manufacturing process. This issue is pivotal in cell therapy treatments, where the dose is determined by the total number of cells administered to a patient. Typically, the manufacturing process involves extracting cells from a patient to expand them into the billions to infuse them back into the patient. However, if the expansion does not reach the target number of cells, the patient may not receive their assigned dose level. This challenge is unique to cell therapies and disrupts the conventional approach of phase I cancer trials, which presuppose that each patient can receive their assigned dose. The essential hurdle lies in effectively utilizing data from patients who receive doses different from their assigned dose, aiming to identify a feasible maximum tolerated dose combination (FMTDC) at the conclusion of the study.

The second challenge arises from the dual escalation of multiple study drugs. Unlike in single-agent dose-finding, where we usually assume toxicity increases with dose level, in multiple-agent dose-finding we may only know a partial ordering of DLT probabilities of the dosecombinations in the study. This complexity is due to the unpredictable joint toxicity effects of the drugs, making it unclear which drug’s increase will result in higher chance of a DLT. To navigate this challenge, we continuously update our estimation of the DLT probability order throughout the trial using newly observed DLT data.

The third challenge arises when DLTs may not happen until well after a patient has begun treatment. For example, some monoclonal antibody treatments can cause late-onset toxicities [4]. Methods to account for these late-onset toxicities are growing more important as cancer research moves past modalities like chemotherapy where toxicity happens within the first cycle of treatment. Furthermore, delays in the observance of DLTs could lead to an underestimation of the drug’s toxicity, potentially resulting in the recommendation of more toxic doses for subsequent study phases. The FDA often approves therapeutics at doses that differ from early-phase study recommendations (Roda et al. [24]), with patients receiving higher, more toxic doses throughout the drug discovery process. To mitigate this, trial designs could extend enrollment periods to allow for the observation of longer toxicity windows. However, this approach slows down patient accrual, which is detrimental both to individuals awaiting enrollment and to the broader population in need of effective treatments, given the already lengthy drug development timeline. In the context of drug-combination cell therapy trials, we tackle this issue by employing statistical models that incorporate pending safety data, allowing for continuous patient enrollment, thereby improving the efficiency of early phase trial design.

Despite the challenges presented, the 3 + 3 algorithm, designed for single-agent studies where DLTs are observed early in treatment, remains ubiquitous in phase I cancer clinical trials [5,6]. It is a particularly poor design for use in cell therapy where simulation studies have shown its potential to ignore large swaths of patient DLT data in its dose-escalation decisions (Bagley and Wages [21]). Practically, it is ill-suited for use in phase I trials that don’t fit neatly into the paradigm developed for investigating single-agent chemotherapies. A phase I cell therapy trial that implemented a 3 + 3 [7] states that “Patients whose chimeric antigen receptor (CAR) T-cell product did not meet the dose to which they were assigned did not inform dose-escalation but were assessed for toxicity and all other parts of the study.” This means that safety data from participants treated at doses different from their assigned levels are excluded from the dose-escalation process, potentially skewing the maximum tolerated dose (MTD) determination. Ignoring safety data from those receiving unplanned dose levels could lead to inaccurate MTD estimates, necessitate a larger sample size, and increase study costs.

Currently, there are limited options for designing and implementing phase I cell therapy trials that address dose-feasibility. Existing designs, such as those by Thall et al. [8] and Wages and Fadul [9], specifically address dose-feasibility issues but are confined to single-agent doseescalation in which late-onset DLTs are not a concern. Bagley and Wages [10] address dose-feasibility with late-onset DLTs, but this is also limited to the single-agent setting. Devlin, Iasonos and O’Quigley [11] introduced a design for cell therapy trials that, while not directly modeling dose-feasibility, adapts the continual reassessment method (CRM) to accommodate intra-dose level assignments in a single-agent context. Despite numerous designs for multi-drug dose-escalation [12–15,23], none confront the unique dose-feasibility challenges posed by cell therapies. This paper introduces a novel dose-finding design, inspired by current real-world dose-finding challenges, that addresses dosefeasibility and late-onset DLTs for drug combination trials.

**Fig. 1.**
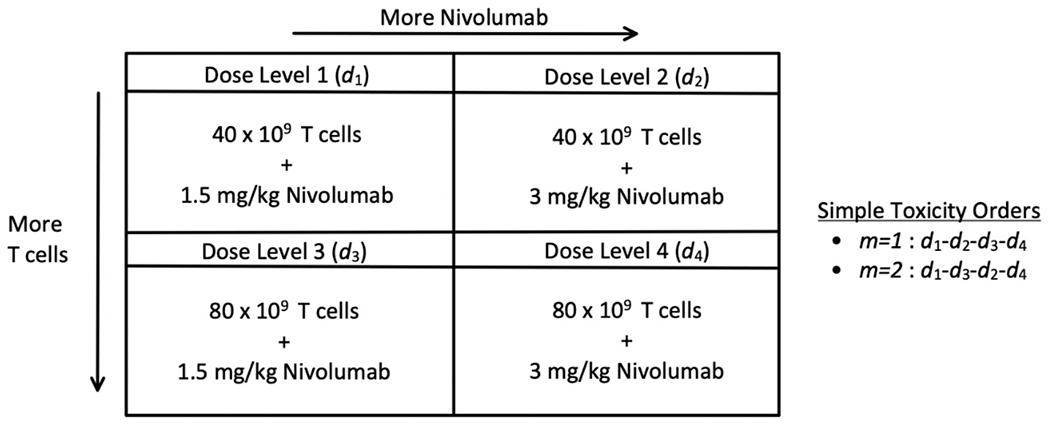
Dose level definitions and simple toxicity orders.

The motivating trial for this work was proposed by investigators at the University of Virginia Cancer Center. It was a dose-escalation trial to investigate Rituximab-based Bispecific antibody activated T cells in combination with Nivolumab for the treatment of high-grade B-cell lymphoma. The T-cells are the cell therapy where some dose levels may not be feasible, and the Nivolumab is an FDA approved immunotherapy where DLTs could happen long after treatment initiation. The dose levels under consideration for both study drugs are given in Fig. 1.

Cell therapy manufacturing starts approximately 2 weeks prior to the patient receiving drug. They receive alternating weekly doses of T-cells and nivolumab over an 8-week period. Treatment related DLTs can occur up to 10 weeks after treatment initiation, thus the DLT observation window is 10 weeks. Further, the toxicity order of the dose levels is not fully known. In this example, two possible orderings exist: *d*_1_ →*d*_2_ →*d*_3_→*d*_4_ and *d*_1_ →*d*_3_ →*d*_2_→*d*_4_. This is because dose levels 2 and 3 have different levels of cell therapy dose and Nivolumab dose whereas dose levels 1 and 4 are defined by combinations with the lowest and highest dose of both drugs respectively. We will present a trial design that accounts for all three of the contemporary dose-finding issues described above by utilizing all available data to execute the dose-escalation process in the most efficient manner possible. In the methodology section we present the methodological framework and mathematical models that guide dose-escalation and FMTDC determination. In the results section, we provide a simulated trial that implements our design and evaluates its operating characteristics over a range of possible clinical scenarios. Lastly, we conclude with a discussion of the design.

## 2. Methodology

Our approach is to assign patient dose levels by modeling toxicity and feasibility using accumulating data from previous patients. To model toxicity, we propose a design framework that extends the methodology by Bagley and Wages [10] into the drug-combination setting. We handle the problem of partially ordered dose levels in a manner that relies upon the partial order continual reassessment method (POCRM) proposed by Wages et al. [14,15]. The method accounts for late-onset DLTs by leaning on the time to event CRM proposed by Cheung and Chappell [16]. All of these methods are reliant, at least in part, on the original CRM proposed by O’Quigley et al. [17].

### 2.1. Toxicity model

We propose a DLT probability model that adaptively assigns patients to one of a set of pre-specified drug combination dose levels where the DLT probability order is only partially known. To address the problem of partially ordered dose levels, we assume there are *m* = 1*,*…,*M* simple dose orders. Further, we model the DLT probability *p_j,m_* at dose level *j* under ordering *m* using a single parameter probit model

(1) $$p_{j,m}\left(\beta ,q_{j,m}\right)=\Phi \left\{\beta +\Phi^{-1}\left(q_{j,m}\right)\right\}\;\mathrm{for}\;j=1,\ldots ,J$$

where Φ(⋅) is the standard normal cumulative distribution function, $\Phi^{-1}(\cdot )$ is the standard normal quantile function, *β* is the parameter of interest and *q_j,m_* are the skeleton values corresponding to a simple order *m* = 1*,*…*, M*. The skeleton can be interpreted as initial DLT probabilities input into the model. We determine the values of the skeleton using the method described by Lee and Cheung [18], as it has been evaluated under extensive simulation scenarios and shown to have robust operating characteristics.

Generally, we assume that the drug will be given throughout a predefined DLT evaluation window, denoted as *T*. A DLT is defined as any adverse event that meets the specific criteria set by the protocol for DLT, and that occurs during this evaluation timeframe. We also assume that a patient that experiences a DLT has met the study endpoint and is considered fully observed. By applying a toxicity model that accommodates the observation of partial DLT data, the status of a patient’s DLT can be understood as varying over time. Let the DLT status of the *i*^th^ patient at any given time, *t*, be defined as

(2) $$y_{i,t}=\left\{\begin{array}{c} 1\;\text{if patient has DLT prior to}\;t\\ 0\;\text{ if patient does not have DLT prior to}\;t \end{array}\right.\mathrm{for}\;i=1,\ldots ,n.$$

The likelihood function under order *m* for the first *n* patients at time *t* is

(3) $$L_{t,m}\left(\beta |y_{i,t},w_{i,t}\right)=\prod_{i=1}^{n}{\left(w_{i,t}p_{j,m}\left(\beta ,q_{j,m}\right)\right)}^{y_{i,t}}{\left(1-w_{i,t}p_{j,m}\left(\beta ,q_{j,m}\right)\right)}^{1-y_{i,t}}$$

where *w_i,t_* is a weight that represents the amount of information patient *i* contributes to the likelihood function. To specify the weights, we implement a linear weight function

(4) $$w_{i,t}=\left\{\begin{array}{c} \frac{t}{T}\;\text{if}\;t<T\\ 1\;\text{if}\;t\geq T\;\text{or}\;y_{i,t} \end{array}\right.\mathrm{for}\;i=1,\ldots ,n.$$

The use of weight functions in the time to event setting was originally described by Cheung and Chappell [16]. Bagley and Wages [10] implemented weight functions to account for late-onset DLTs in the context of cell therapies where dose-feasibility was an issue for singleagent escalation. No methods have used weight functions to account for late-onset toxicities where both dose-feasibility and toxicity order must be taken into consideration.

To select a particular order *m,* we weight each of the orders by

(5) $$\omega (m|\mathrm{data})=\frac{\omega \left(m\right)\int L_{t,m}\left(\beta |y_{i,t},w_{i,t}\right)f\left(\beta \right)d\beta }{\sum_{m=1}^{M}\omega \left(m\right)\int L_{t,m}\left(\beta |y_{i,t},w_{i,t}\right)f\left(\beta \right)d\beta }\mathrm{for}\;m=1,\ldots ,M$$

where *ω*(*m*) is the prior probability that order *m* is the correct order. The order with the largest posterior probability *ω*(*m*|data) is, according to the data, most likely to be the correct order. In general, we assume *ω*(*m*) to have a discrete uniform distribution, but if prior information is available that suggests a particular order the prior distribution can be weighted to reflect this information. We calculate the weights each time a patient is accrued to trial so they will be assigned a dose level under the order with largest posterior probability, breaking ties at random. To illustrate this, consider the hypothetical patient data given in Table 1, where the possible dose orders are *m* = 1: *d*_1_ – *d*_2_ – *d*_3_ – *d*_4_*,* and *m* = 2: *d*_1_ – *d*_3_ – *d*_2_ – *d*_4_*.*It is unknown whether *d*_2_ is more or less toxic than *d*_3_ so a movement from *d*_2_ to *d*_3_ does not necessarily represent a dose-escalation decision.

The observed DLT rates at *d*_2_ and *d*_3_ are 2/3 and 1/3 respectively. This is evidence that *d*_2_ could be more toxic than *d*_3_. Evaluating eq. (5) for both orders under this data set yields *ω*(*m* = 1|data) = 0.39 and *ω*(*m* = 2|data) = 0.61. Thus, when patient 8 is enrolled to the trial we would assign their dose level assuming *m* = 2 is the correct order since it has the largest posterior probability. This should make intuitive sense because we observe a higher DLT rate for *d*_2_ than *d*_3_, and order *m* = 2 aligns with this belief. We note that, for simplicity, Table 1 assumes that each patient has completed their DLT observation window, but this need not be the case generally. Wages, Conaway and O’Quigley (2011,2013) use a similar method to select the dose orders, but their methods were proposed and evaluated in a setting where dose-feasibility issues were not a concern.

Taking eqs. (1)–(5) together, we can estimate the DLT probability at each dose level, under a selected order *m*, at time *t* as

(6) $${\hat{p}}_{j,m}=\int \frac{\Phi \left\{\beta +\Phi^{-1}\left(q_{j,m}\right)\right\}L_{t,m}\left(\beta |y_{i,t},w_{i,t}\right)f(\beta )d\beta }{\int L_{t,m}\left(\beta |y_{i,t},w_{i,t}\right)f(\beta )d\beta }\mathrm{for}\;j=1,\ldots ,J.$$

The estimated MTDC under order *m* at time *t* is

(7) $$j_{t,m}^{*}={argmin}_{j}\left|{\hat{p}}_{j,m}-\theta^{*}\right|\;\mathrm{for}\;j=1,\ldots ,J,$$

where θ* is the prespecified target DLT rate that defines the MTDC. Using these definitions, under a particular order *m*, we define the set of safe dose levels as all doses with DLT probability less than or equal to θ*. Mathematically this set of dose levels is defined as

(8) $${𝒮}_{m}=\left\{d_{j}:{\hat{p}}_{j,m}\leq {\hat{p}}_{j^{*},m}\right\}$$

where *m* is a particular simple order and ${\hat{p}}_{j,m}$ is the estimated DLT probability of the MTD defined in (7). This set, along with the toxicity modeling framework will be used in conjunction with the feasibility model, presented in the subsequent section, to guide dose selection and patient enrollment.

To ensure patient safety we implement a toxicity stopping rule so that patients will not be exposed to overly toxic dose levels. This rule is based on the model we have developed and halts the trial if the lowest dose level becomes too toxic. We stop the trial if

(9) $$P(\text{dose level}\;1\;\text{is too toxic }|\text{ data })=\text{P}\left(\beta >{\text{Φ}}^{-1}\left(\theta^{*}\right)-{\text{Φ}}^{-1}\left(q_{1}*\right)|\text{ data }\right)>p_{u,t}$$

where *p_u,t_* is an upper probability cutoff for toxicity. If at any point the toxicity stopping rule is triggered the trial halts and the FMTDC is set to 0.

### 2.2. Feasibility model

When a patient enrolls in the trial and has their cell therapy product manufactured, each patient will have a set of dose levels they are feasible to receive. Let the dose levels of the cell therapy be indexed by *k* = 1,…,*K* where *K* is the total number of cell therapy doses. For example, for the dose levels defined in Fig. 1, cell therapy dose *k* = 1 corresponds to dose levels *d*_1_ and *d*_2_ and *k* = 2 corresponds to dose levels *d*_3_ and *d*_4_. Consequently, each specific value of *k* corresponds to a set of dose levels that are equally feasible to receive. A patient’s individual highest feasible cell dose (IHFCD) is defined as the largest cell therapy dose *d_k_* a patient could receive based upon the number of cells expanded during manufacturing. Patients’ IHFCDs are recorded as a count in the multinomial vector

(10) $$X=\left(X_{0},X_{1},\ldots ,X_{K}\right)∼Multinomial\left(\pi_{0},\pi_{1},\ldots ,\pi_{K}\right)$$

where $\pi =\left(\pi_{0},\pi_{1},\ldots ,\pi_{K}\right)$ are the probabilities corresponding to each component of ***X*** and $\sum_{k=0}^{K}\pi_{k}=1$. For example, if a patient’s IHFCD is *k* = 2 they are feasible to receive dose levels *d*_1_*, d*2*, d*3*,* and *d*_4_ and their IHFCD is indexed in *X*_2_. Alternatively, if a patient’s IHFCD is *k* = 1 they are feasible to receive dose levels *d*_1_and *d*_2_*,* their IHFCD is indexed in *X*_1_. In the event a patient is not feasible to receive the lowest defined cell therapy dose (*k* = 0), they are indexed in *X*_0_. If a patient is indexed in *X*0 they are not treated at one of the predefined dose levels and thus are not included in the toxicity model.

We are primarily interested in determining which set(s) of dose levels are feasible across the patient population to determine the FMTDC. To do this, we first need to determine the global highest feasible cell dose (GHFCD), defined as the estimated cell dose that a prespecified proportion of the patients can receive. Let ***π*** ~ Dirichlet(**a**), where $=\left(a_{0},a_{1},\ldots ,a_{K}\right)$ is the parameter vector for the Dirichlet distribution. We note the conjugate family relationship gives the posterior distribution as π∣X~Dirichlet(a+X).

The posterior probability that cell dose *k* is feasible can be written as the sum

(11) $$\varphi_{k}=\pi_{k}+\pi_{k+1}+\ldots +\pi_{K}.$$

Dose level *d_j_* is globally feasible, if its associated cell dose *k* satisfies

(12) $$P\left(\varphi_{k}<\varphi^{*}|X\right)<p_{u,f}$$

where φ* is the minimum required feasibility probability, and *p_u,f_* is an upper probability cutoff. The value for φ* is protocol specific, but we would expect it to be relatively large (≥0.50) to reflect a sufficient threshold for dose-feasibility. For instance, in the motivating trial, and the trial described by Wages and Fadul [9] it is specified to be 0.80. Values for *p_u,f_* should be large (≥0.90) and tuned via simulation so that the feasibility stopping rule is only triggered when we are very sure the lowest dose level is not feasible.

The probabilities can be calculated from the posterior distribution $\varphi_{k}|X∼Beta\left(\sum_{r=k}^{K}a_{r}+X_{r},\sum_{\ell =0}^{k-1}a_{\ell }+X_{\ell }\right)$. The GHFCD is defined as

(13) $${\hat{d}}_{k}=\underset{k\in \{1,\ldots ,K\}}{max}\left\{d_{k}:P\left(\varphi_{k}<\varphi^{*}|X\right)<p_{u,f}\right\}.$$

Then define the function

(14) $$\mu \left(d_{v}\right)=\text{number of cells defined by dose}{\;d}_{v}\text{.}$$

Finally, taking all of this together the set of globally feasible dose levels is defined as

(15) $${ℱ}_{k}=\{d_{j}:\mu (d_{j})\leq \mu \left({\hat{d}}_{k}\right)\}.$$

In other words, any dose that is defined by a cell dose level likely to be feasible across a patient population is a member of this set. If the lowest cell dose is not in ${ℱ}_{k}$ we stop the trial early due to insufficient feasibility. The feasibility models discussed in this section are similar to those in Thall, Sung and Choudhury [8] and Bagley and Wages [10], but those models were designed for single-agent dose-escalation, whereas the proposed models are designed for multi-drug dose-escalation.

Now that we have defined the set of safe dose levels ${𝒮}_{m}$, and the set of globally feasible dose levels ${ℱ}_{k}$, we define the set of dose levels that are both feasible and safe as

(16) $$\Omega_{SF}={𝒮}_{m}\cap {ℱ}_{k}$$

where *m* is the selected order under all observed data. From this we define the FMTDC as

(17) $$FMTDC=\underset{j\in \Omega_{SF}}{arg\;min}\left|{\hat{p}}_{j,m}-\theta^{*}\right|$$

where ${\hat{p}}_{j,m}$ are the estimated DLT probabilities under model *m* at the conclusion of the trial. If the trial stops early due to the lowest dose level being overly toxic or insufficiently feasible, the FMTDC is set to 0.

### 2.3. Trial conduct

Table 2 describes the algorithm that governs trial conduct and dose assignment.

## 3. Results

### 3.1. Application of design to a simulated trial

**Fig. 2.**
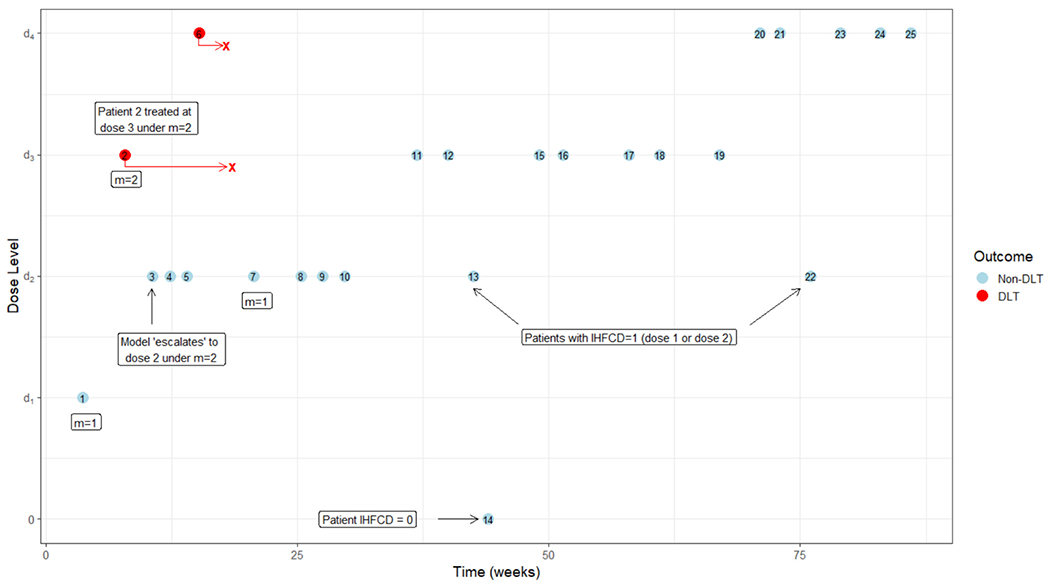
Simulated trial guided by proposed design. Labels m =1 or m =2 indicate points in time when the estimated toxicity order changes An additional simulated trial is given in the supplemental appendix (Fig. S2).

To show how our design would work in practice we provide a simulated trial depicted in Fig. 2. This simulation illustrates how the design adaptively adjusts to the partial ordering of DLT probabilities while accounting for late-onset toxicities and patients with dose-feasibility limitations. Simulation settings were chosen to conform to the motivating trial for this design. The dose levels under consideration are defined in Fig. 1. The target DLT rate is θ* = 0.25, the minimum targeted feasibility rate is φ* = 0.80, and the upper probability cutoffs are *p_u,f_* = 0.90 and *p_u,t_* = 0.90. The maximum number of patients to be evaluated for toxicity under the protocol is *N* = 24 with a maximum of *C* = 30 patients to have their cells expanded and evaluated for dose-feasibility. The DLT evaluation window is 10 weeks, and patient time to toxicity are drawn from a uniform(0, 10) distribution. It is assumed that an average of 1 patient arrives every 4 weeks according to a Poisson process. We model partially observed DLT probabilities using the linear weight function described in eq. (4). The skeleton is set to (0.13, 0.25, 0.41, 0.59) following the algorithm described by Lee and Cheung [18]. To specify the prior distribution on *β* we use the least informative normal prior *N* (0, 0.74^2^) defined by Lee and Cheung [19]. We used these prior specifications as they have been shown to have robust operating characteristics across a large variety of simulation scenarios. Recall that the two DLT orders are *d*_1_ – *d*_2_ – *d*_3_ – *d*_4_ for *m* = 1 and *d*_1_ – *d*_3_ – *d*_2_ – *d*_4_ for *m* = 2. Prior to the trial we do not have any indication of which order is more likely, so we set the prior probabilities of the orders to *ω*(*m* = 1) = *ω*(*m* = 2) = ½. The prior for the feasibility model was specified as a discrete uniform. This selection was guided by previous work (Bagley and Wages [21]) showing superior operating characteristics across many scenarios. We also impose that the trial must start at the lowest dose level, and that skipping untried dose levels based on the current order is not allowed.

The simulated trial, given in Fig. 2, begins with order *m* = 1 selected at random. Patient 1 is treated at dose level 1 since the trial must start at this dose level. By the time patient 2 enrolls in the trial, we still have no toxicity order information, so we select the order *m* = 2 at random, and the dose with estimated DLT probability closest to θ* is dose level 3 so that is what patient 2 receives. When patient 3 enrolls in the trial, patient 2 has not had their DLT yet, but the order with highest posterior probability is *m* = 2 and the estimated DLT probabilities for *d*_2_ and *d*_3_ are 0.30 and 0.15 respectively so patient 3 receives dose level 2. For the next two patients the order is *m* = 2 and they receive dose level 2. When patient 6 enrolls in the trial, dose level 4 has estimated DLT probability closest to the target, so they receive that dose. By the time patient 7 enrolls in the trial, both patient 2 and patient 6 have had their DLTs and the posterior probability for each order are 0.75 and 0.25 for *m* = 1 and *m* = 2 respectively. Thus, for the rest of the trial, since there are no more DLTs, *m* = 1 has the highest posterior probability. Patients 7–10 receive dose level 2, but by the time patient 11 enrolls the estimated DLT probability for dose 3 is closest to the target DLT rate so patients 11 and 12 receive dose level 3. Patients 13 and 14 are assigned to receive dose level 3, but they do not have enough cells expanded to be treated at this dose level. Patient 13 receives their IHFCD (dose 2), and patient 14 is excluded from the trial as they did not have enough cells expanded to reach even the lowest dose level. The feasibility stopping criteria is checked at this point, but all doses are still in the globally feasible set. The trial proceeds with patients 15–19 allocated to dose level 3 since that is the estimated MTD. Patients 20–25 are assigned to receive dose level 4, but patient 22 receives dose level 2 due to feasibility issues. At the conclusion of the trial, dose 4 is selected as the FMTDC under order *m* = 1 since it has an estimated DLT probability that is closest to the target DLT rate, and the globally feasible set is ${𝒡}_{k}=\left\{d_{1},d_{2},d_{3},d_{4}\right\}$.

### 3.2. Simulation study

To evaluate the proposed methodology, we conducted Monte-Carlo simulations across 12 pairs of randomly generated toxicity and feasibility curves. Unless otherwise noted, simulation settings for this study were selected to match those described in the simulated trial in the previous section. The DLT curves are “s” shaped curves generated by the algorithm given by Conaway and Petroni [20]. These curves were generated in the single-agent dose-escalation framework, so they are monotonically increasing. For the scenarios 1–6 we left the curves as is, thus *m* = 1 is correct. For scenarios 7–12 we reversed the DLT probabilities for *d*_2_ and *d*_3_ so *m* = 2 is the correct choice. To generate feasibility scenarios, we used the algorithm described by Bagley and Wages (2021 [21]). In this class of curves feasibility probability decreases as the cell dose increases. Since feasibility curves do not depend on DLT order, scenarios 1–6 match scenarios 7–12 for comparisons sake. FMTDC selection percentages are given in Table 3. For each scenario, we conducted 2000 simulated trials.

For the first two scenarios, the true FMTDC are dose levels 1 and 2 respectively and the design successfully selects these dose levels most of the time as the recommended FMTDC. The correct selection decreases in scenarios 3 and 4, likely due to the closeness of the DLT probabilities of dose levels 2,3, and 4. Scenario 5 is a situation where the lowest dose level is overly toxic, thus the trial ends early for most of the trials. Scenario 6 has no sufficiently feasible dose levels, so it also correctly stops nearly 90 % of the time. For scenarios 7–12, the correct order is *m* = 2. For scenario 7, the FMTDC percentage increases by 12 % over scenario 1. This is likely due to a lack data being observed at *d_2_* due to low feasibility. Scenario 8 is interesting because the true FMTDC changes from *d_2_* to *d_1_* due to low feasibility. This increases the PCS of the FMTDC because the model can adjust to the true dose order and *d_2_*is too toxic. Scenarios 9 and 10 perform similarly to scenarios 3 and 4 since there aren’t large feasibility issues and the model adjusts to the different DLT order. Scenarios 11 and 12 aren’t much different from scenarios 5 and 6 since the correct decision is to stop the trial early.

Our simulations show that our design has good operating characteristics in this drug combination setting. Comparing our operating characteristics to other designs’ is difficult because there are no designs that address dose-feasibility, drug combinations, and late-onset DLTs. Only 2 other published methods target an FMTD [8–10] and those are only in the single-agent dose-escalation setting. We have included further simulation results for additional scenarios in Table S1 in the supplemental appendix.

## 4. Discussion

An FDA guidance document (2024 [22]) focusing on the development of cell therapy products underscores the importance of understanding potential manufacturing failures from the outset of early-phase trials. The document states that, “It is important to gain an understanding from early-phase trials of the likelihood of manufacturing failure…” and suggests that “Failure-to-treat may be an important trial endpoint that is part of a feasibility evaluation…” The FDA is advocating for a thorough inclusion of feasibility evaluations in the trials for cell therapy products.

We have developed the first, and currently only, methodology for early phase drug combination trials tailored specifically to address cell therapy products that may encounter dose-feasibility issues. This innovative design is structured to allow for the efficient inclusion of late-onset DLT data and to manage multiple toxicity orders. Our method provides a more nuanced understanding of how issues within drug combination trials may interact with issues in the specific context of cell therapies.

The objective of our approach is to enable clinical researchers to conduct early-phase trials in a more efficient manner when it comes to cell therapeutics. By improving the design and execution of these early-phase trials, we hope to contribute to a smoother and more efficient pathway through the complex landscape of drug development, ultimately reducing the time it takes to bring effective and safe cell therapy products to patients. In this spirit, we have provided code for our trial design a publicly available github repository accessible at https://github.com/trialcan/feasibility-in-drug-combos.git.

## Supplementary Material

1

## Figures and Tables

**Table 1:** Hypothetical patient DLT data.

Patient #	1	2	3	4	5	6	7
Dose Received	*d*_1_	*d*_2_	*d*_2_	*d*_2_	*d*_3_	*d*_3_	*d*_3_
DLT status	0	1	1	0	0	0	1

**Table 2:** Trial conduct algorithm.

Step	Procedure
1	Accrue patient to the study
2	Feasibility determination •Determine Patient’s IHFCD. •If IHFCD >0 proceed to step 3. •If IHFCD = 0: ⚬ Compute GHFCD (eq. 13) ⚬ If GHFCD = 0: ▪ Stop trial, set FMTDC = 0. ⚬ Else (GHFCD>0). ▪ Return to step 1.
3	Toxicity evaluation •Determine patient’s toxicity outcomes and update DLT data. •If toxicity stopping rule is triggered (eq. 9): ⚬ Stop the trial and set FMTDC = 0. •Else, proceed to step 4.
4	Select Dose Order •Calculate posterior probability of toxicity orders (eq. 5). •Select order with largest posterior probability resolving ties randomly. •Under this order, update estimated DLT probabilities (eq. 6). •Proceed to step 5.
5	Assign Dose and Continue Enrollment •If current patient IHFCD ≥ j*_t,m_ (eq. 7). ⚬ Treat patient at j*_t,m_ •Else, ⚬ Treat at patients the highest feasible dose with DLT probability closest to the target that does not exceed MTDC’s DLT probability. •If trial is not fully enrolled, return to Step 1.
6	Complete patient follow-ups and make final estimates •Complete patient follow-ups. •Estimate final FMTDC (eq. 17).

**Table 3:** FMTDC% is the percentage of trials that selected the indicated dose level as the FMTDC. A bold value indicates a correct selection or the percentage of correct selection (PCS). True DLT and feasibility probabilities are represented by p_t_ and p_f_ respectively. The column labeled ‘None’ indicates that no FMTDC was selected due to the trial stopping early due to the feasibility or toxicity stopping rules.

Scenario	Dose		*p_t_*		*p_f_*	FMTDC %		None
1	*d*_1_	*d*_2_	0.22	0.32	0.89	**58.5**	36.8	4.7
	*d*_3_	*d*_4_	0.38	0.46	0.37	0.0	0.0	
2	*d*_1_	*d*_2_	0.18	0.24	0.87	34.8	**60.4**	4.8
	*d*_3_	*d*_4_	0.36	0.40	0.36	0.0	0.0	
3	*d*_1_	*d*_2_	0.10	0.15	0.96	3.0	34.8	0.1
	*d*_3_	*d*_4_	0.25	0.37	0.91	**44.5**	17.6	
4	*d*_1_	*d*_2_	0.11	0.15	0.97	2.5	33.5	0.1
	*d*_3_	*d*_4_	0.19	0.29	0.84	34.9	**29.0**	
5	*d*_1_	*d*_2_	0.50	0.58	0.96	32.2	0.8	**66.7**
	*d*_3_	*d*_4_	0.64	0.78	0.88	0.3	0.0	
6	*d*_1_	*d*_2_	0.31	0.37	0.60	6.9	1.5	**91.6**
	*d*_3_	*d*_4_	0.63	0.88	0.23	0.0	0.0	
7	*d*_1_	*d*_2_	0.22	0.38	0.89	**70.7**	23.7	5.7
	*d*_3_	*d*_4_	0.32	0.46	0.37	0.1	0.0	
8	*d*_1_	*d*_2_	0.18	0.36	0.87	**65.6**	29.0	5.6
	*d*_3_	*d*_4_	0.24	0.40	0.36	0.1	0.0	
9	*d*_1_	*d*_2_	0.10	0.25	0.96	3.7	**44.1**	0.1
	*d*_3_	*d*_4_	0.15	0.37	0.91	36.5	15.7	
10	*d*_1_	*d*_2_	0.11	0.19	0.97	2.7	39.5	0.1
	*d*_3_	*d*_4_	0.15	0.29	0.84	29.6	**28.3**	
11	*d*_1_	*d*_2_	0.50	0.64	0.96	32.0	0.6	**66.9**
	*d*_3_	*d*_4_	0.58	0.78	0.88	0.6	0.0	
12	*d*_1_	*d*_2_	0.31	0.63	0.60	6.8	0.1	**93.2**
	*d*_3_	*d*_4_	0.37	0.88	0.23	0.0	0.0	

## Data Availability

No data was used for the research described in the article.

## Appendix A. Supplementary data

Supplementary data to this article can be found online at https://doi.org/10.1016/j.cct.2025.107894.
