# Supplementary material for: Dose-finding designs for cell therapy cancer clinical trials evaluating drug-combinations
Source: Contemp Clin Trials. Author manuscript; Available in PMC 2026 May 27. (PMC13213886; doi:10.1016/j.cct.2025.107894)
Supplement: 1 [file NIHMS2173373-supplement-1.docx]

Table S1: Additional Simulation Results

| Scenario | Dose | | *p_t_* | | *p_f_* | FMTD % | | None |
| --- | --- | --- | --- | --- | --- | --- | --- | --- |
| S1 | *d*_1_ | *d*_2_ | 0.10 | 0.15 | 0.89 | 11.6 | **86.3** | 2.2 |
|  | *d*_3_ | *d*_4_ | 0.25 | 0.37 | 0.37 | 0.1 | 0.0 |  |
| S2 | *d*_1_ | *d*_2_ | 0.11 | 0.15 | 0.87 | 13.6 | **82.1** | 4.4 |
|  | *d*_3_ | *d*_4_ | 0.19 | 0.29 | 0.36 | 0.0 | 0.0 |  |
| S3 | *d*_1_ | *d*_2_ | 0.22 | 0.32 | 0.70 | 20.8 | 15.1 | **63.6** |
|  | *d*_3_ | *d*_4_ | 0.38 | 0.46 | 0.50 | 0.6 | 0.0 |  |
| S4 | *d*_1_ | *d*_2_ | 0.18 | 0.24 | 0.70 | 13.5 | 25.7 | **60.2** |
|  | *d*_3_ | *d*_4_ | 0.36 | 0.40 | 0.50 | 0.5 | 0.1 |  |
| S5 | *d*_1_ | *d*_2_ | 0.10 | 0.15 | 0.70 | 5.3 | 34.7 | **59.0** |
|  | *d*_3_ | *d*_4_ | 0.25 | 0.37 | 0.50 | 0.5 | 0.6 |  |
| S6 | *d*_1_ | *d*_2_ | 0.11 | 0.15 | 0.70 | 6.0 | 32.2 | **60.2** |
|  | *d*_3_ | *d*_4_ | 0.19 | 0.29 | 0.50 | 1.1 | 0.6 |  |
| S7 | *d*_1_ | *d*_2_ | 0.50 | 0.58 | 0.70 | 12.3 | 0.4 | **87.3** |
|  | *d*_3_ | *d*_4_ | 0.64 | 0.78 | 0.50 | 0.1 | 0.0 |  |
| S8 | *d*_1_ | *d*_2_ | 0.31 | 0.37 | 0.70 | 26.1 | 7.8 | **66.2** |
|  | *d*_3_ | *d*_4_ | 0.63 | 0.88 | 0.50 | 0.0 | 0.0 |  |
| S9 | *d*_1_ | *d*_2_ | 0.22 | 0.32 | 0.50 | 0.5 | 0.8 | **98.8** |
|  | *d*_3_ | *d*_4_ | 0.38 | 0.46 | 0.30 | 0.0 | 0.0 |  |
| S10 | *d*_1_ | *d*_2_ | 0.18 | 0.24 | 0.50 | 0.2 | 0.7 | **99.2** |
|  | *d*_3_ | *d*_4_ | 0.36 | 0.40 | 0.30 | 0.0 | 0.0 |  |
| S11 | *d*_1_ | *d*_2_ | 0.10 | 0.15 | 0.50 | 0.1 | 1.0 | **98.9** |
|  | *d*_3_ | *d*_4_ | 0.25 | 0.37 | 0.30 | 0.0 | 0.0 |  |
| S12 | *d*_1_ | *d*_2_ | 0.11 | 0.15 | 0.50 | 0.0 | 0.9 | **99.1** |
|  | *d*_3_ | *d*_4_ | 0.19 | 0.29 | 0.30 | 0.0 | 0.0 |  |
| S13 | *d*_1_ | *d*_2_ | 0.50 | 0.58 | 0.50 | 0.6 | 0.0 | **99.4** |
|  | *d*_3_ | *d*_4_ | 0.64 | 0.78 | 0.30 | 0.0 | 0.0 |  |
| S14 | *d*_1_ | *d*_2_ | 0.31 | 0.37 | 0.50 | 0.9 | 0.2 | **99.0** |
|  | *d*_3_ | *d*_4_ | 0.63 | 0.88 | 0.30 | 0.0 | 0.0 |  |


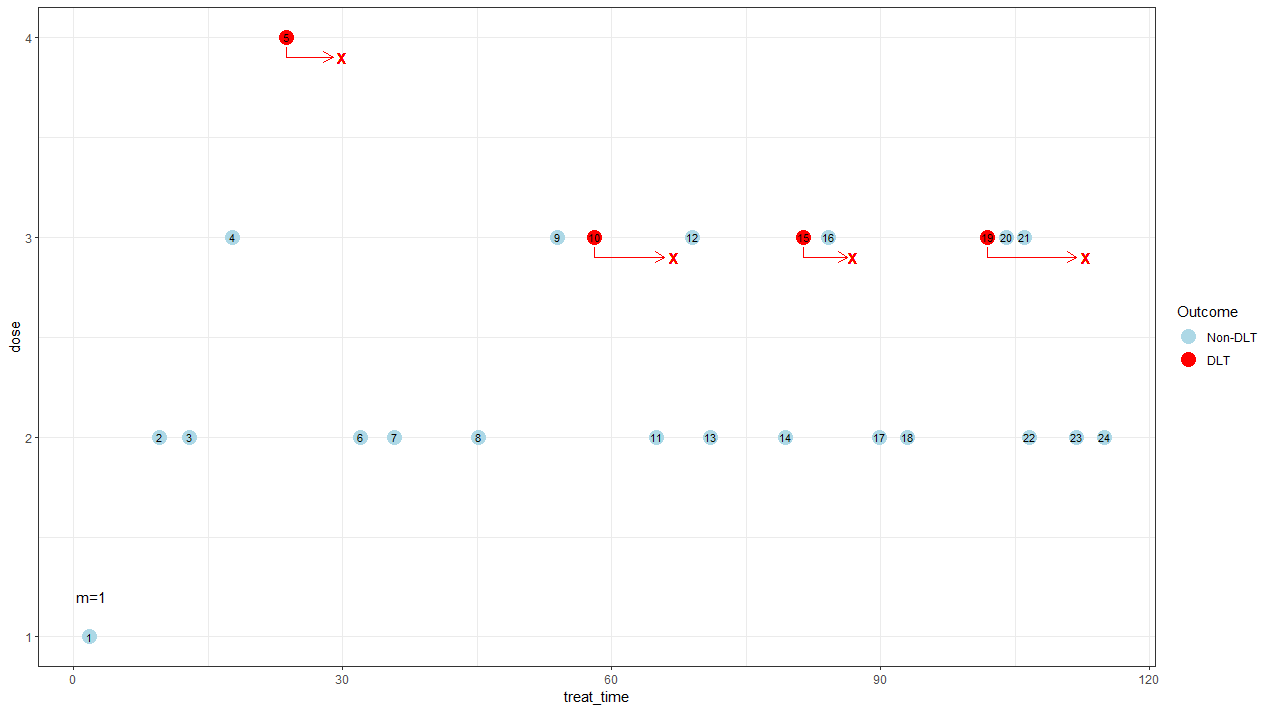


Figure S1 In this simulated trial the dose level with DLT rate closest to the target is dose level 3. Dose level 1 and 2 are highly feasible and safe whereas dose levels 3 and 4 are not globally feasible. Most participants treated at dose level 2 were only feasible to receive dose level 1 or 2. Notably the trial treats patients at dose level 3 when feasible as it is sufficiently safe. At the conclusion of the trial the MTDC is dose level 3, but the globally feasible set only includes dose levels 1 and 2. So the Final FMTDC is dose level 2 under order m=1 (which is the order throughout this trial).
